# AIEgens-Doped Photonic Crystals for High Sensitivity Fluorescence Detection of Tumor Markers

**DOI:** 10.3390/bios13020276

**Published:** 2023-02-15

**Authors:** Zhijun Liao, Qian Zhou, Bingbing Gao

**Affiliations:** School of Pharmaceutical Sciences, Nanjing Tech University, Nanjing 211816, China

**Keywords:** tumor markers, detection, aggregation-induced emission, photonic crystals, fluorescence

## Abstract

Detection of tumor markers is of great significance to preliminarily judge whether patients have malignant tumors. Fluorescence detection (FD) is an effective means to achieve sensitive detection of tumor markers. Currently, the increased sensitivity of FD has attracted research interest worldwide. Here, we have proposed a method of doping luminogens with aggregation-induced emission (AIEgens) into photonic crystals (PCs), which can significantly enhance the fluorescence intensity to achieve high sensitivity in the detection of tumor markers. PCs are made by scraping and self-assembling, which has the special effect of fluorescence enhancement. The combination of AIEgens and PCs can enhance the fluorescence intensity 4–7 times. These characteristics make it extremely sensitive. The limit of detection (LOD) for the detection of alpha-fetoprotein (AFP) in the AIE10 (Tetraphenyl ethylene-Br) doped PCs with a reflection peak of 520 nm is 0.0377 ng/mL. LOD for the detection of carcinoembryonic antigen (CEA) in the AIE25 (Tetraphenyl ethylene-NH_2_) doped PCs with a reflection peak of 590 nm is 0.0337 ng/mL. Our concept offers a good solution for highly sensitive detection of tumor markers.

## 1. Introduction

Cancer is a general term for a series of malignant tumors, causing great damage to the human body; it has a high mortality, needs timely prevention and diagnosis, and its treatment is of great research significance. At present, it is known that there are many diagnostic methods for cancer, and the detection of tumor markers is a common diagnostic method. Compared with other methods, the detection of tumor markers is more convenient, the wound is smaller, the price is relatively low, and only a small part of blood drawn from the patient’s body is required to carry out relevant detection, so the tumor markers are more acceptable to patients. Detection of tumor markers is a significantly used means for the diagnosis of tumors, assessment of efficacy, detection of relapse, and prediction of prognosis [1]. Usually, the examination contents include carcinoembryonic antigen (CEA), alpha-fetoprotein (AFP), prostate specific antigen (PSA), etc. [2]. Frequent detection methods for tumor markers include radioimmunoassay (RIA), FD, fluid biopsy, proteomics, and immunosensor [3]. Among them, FD is an effective method for the detection of tumor markers, which has attracted much attention due to its simple operation, good safety, high stability, high precision and other characteristics [4]. Therefore, the improvement of FD stability has aroused great research interest [5]. Well-known FD methods include nano fluorescent probes, fluorescence immune sensing platform, flow fluorescence immune method, fluorescence imaging [6]. Chen et al. utilized a single nanoparticle containing tens of thousands of rare earth ions to increase the labeling ratio of the analyte, thereby enhancing the sensitivity of fluorescence immunoassay [7]. Our team also conducted some research on the fluorescence enhancement of photonic crystals (PCs) [8]. Quenching occurs so that the adjacent luminophores experience strong intermolecular π-π stacking interaction. The excited states of these aggregates usually decay or relax back to the ground state through non-radiative channels, resulting in emission quenching of luminophores when the fluorescent substance is too thick, and the detection requirement cannot be met when the fluorescent substance is too thin. Therefore, there is an urgent need to develop a FD method capable of detecting tumor markers with high sensitivity [9]. 

Since the luminogens with aggregation-induced emission (AIEgens) does not undergo fluorescence quenching, we can enhance the sensing sensitivity by increasing the concentration [10]. In addition, PCs have unique fluorescence property, and are often used for preparing fluorescence analysis instrument. Therefore, we have attempted to combine the two substances to achieve a highly sensitive detection of tumor markers [11]. Silica PCs are composed of two or more materials with periodic nanostructures. Its unique optical properties and unique nanostructures provide an effective means for biological and chemical analysis [12]. The local confinement of photons by the internal defect layer generates high regional light intensity to enhance fluorescence [13]. Gu et al. developed a sensor for eye health monitoring using the enhanced fluorescence effect of PCs [14]. Immunoassays that incorporate fluorometry are greatly excited by their simplicity, speed, and stability. However, the application of conventional fluoroimmunoassay is generally limited by the quenching phenomenon caused by aggregation. The discovery of AIEgens properties offers new options for fluorescence sensing [15]. The concept of AIEgens was proposed by Tang’ s group in 2001, that substances such as hexaphenylsiloxanes are nonemissive in an isolated molecular state but give strong emissions as aggregates or cluster [16]. Consequently, compared with traditional fluorescent agents, AIEgens usually show higher photobleaching thresholds, excellent photostability and higher signal reliability [17]. The use of AIEgens has generated strong research interest and opened the way to a range of possibilities for FD [18]. The combination of AIEgens and PCs is expected to realize the high sensitivity detection of tumor substances [19]. 

**Figure 1:**
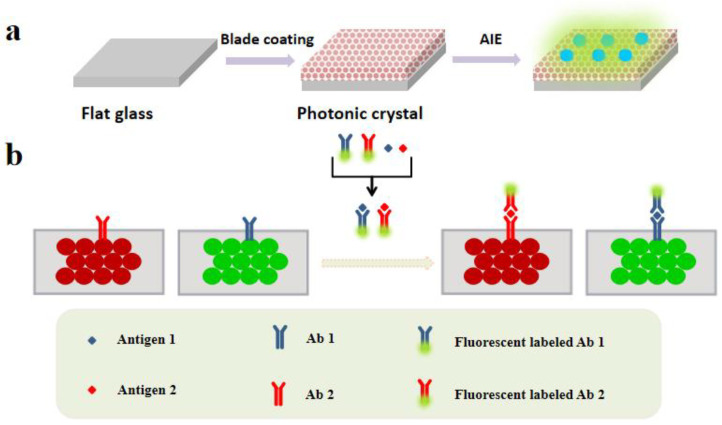
(**a**) Manufacturing process of AIEgens-doped PCs. (**b**) The procedure of multiplex detection based on the optical encoding property of the PCs.

In this paper, the fluorescence enhancement benefit generated by the combination of the PCs and the AIEgens can be used for detecting tumor markers with high sensitivity [20]. As shown in Figure 1a, PCs are manufactured by a scratch coating self-assembly method, and has fluorescence enhancement, and the fluorescence intensity can be enhanced by 4–7 times when being combined with AIEgens. These features allow that method to have a low detection limit. Figure 1b summarizes the principle of this method. After combining AIEgens with antibody, antibody and antigen specific recognition formed a complex, which in turn combined with the antibody on the PCs to form a complex with a quantity of AIEgens. Wash away the AIEgens that are not bound to the PCs with clear water, and the background signal is relatively low, but this AIE molecule can emit a bright signal. The complexes emitting fluorescence can be seen under the fluorescence microscope, and the higher the concentration of antigen, the more complex formed and more bound to PCs, and the greater the fluorescence intensity, so we can reflect the concentration of the test object by detecting the fluorescence intensities, which is of great significance in the detection of tumor markers. To the best of our knowledge, we are the first to report this, which is useful not only in this field but also in the detection of fluorescence sensors, skin diseases and fundus diseases [21]. 

## 2. Results and Discussion

### 2.1. Fabrication of AIEgens-Doped PCs

Preparation of colloidal solution of SiO_2_ nanoparticles (NPs): 3 mL of ammonia water and 29 mL of distilled water were added into 63.5 mL of ethanol, and magnetically stirred for 10 min at room temperature, to obtain solution M. Then a certain amount of ethyl orthosilicate was dissolved in ethanol and stirred for 10 min to obtain solution N. Then, placing the solution M in a heat-collecting constant-temperature oil bath pot at 25 °C, solution N was quickly added into the solution M after the temperature of the solution M was constant. The mixture was magnetically stirred for 8 h to obtain a white emulsion; Subsequently, the samples obtained using the reaction were subjected to centrifugal washing several times and then placed in a vacuum drying oven. The temperature was kept at 80 °C for 12 h until the ethanol was completely volatilized to obtain SiO_2_ with a particle size of 240 nm. SiO_2_ with a particle size of 274 nm could be prepared by changing the reaction temperature to 30 °C. The dried SiO_2_ was collected and prepared into an ethanol solution with a mass fraction of 5%.

**Figure 2:**
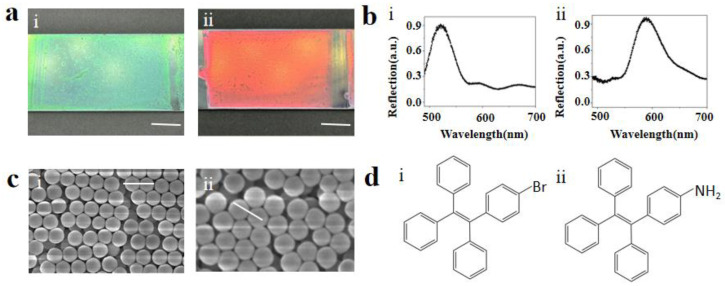
(**a**) PCs of different colors are scraped on flat glass. (**i**) green (**ii**) red, scale bar: 1 cm. (**b**) Reflectance spectra of the corresponding PCs. (**i**) 520 nm (**ii**) 590 nm. (**c**) Scanning electron microscopy images of the PCs. (**i**) Green PCs, scale bar: 500 nm (**ii**) red PCs, scale bar: 500 nm. (**d**) Chemical structure of different AIEgens. (**i**) AIE10 (**ii**) AIE25.

The silicon dioxide crystal template was formed by self-assembly of monodisperse silicon dioxide nanoparticles scratch-coated flat glass. Briefly, the glass was completely cleaned, then the SiO_2_ NPs colloidal solution was dripped on the edge of the glass sheet, and then the glass sheet which was dipped with the solution was used to scrape and coat a new glass sheet. Then, by solvent evaporation, SiO_2_ NPs with diameters of 240 nm and 274 nm were self-assembled into PCs on glass [22]. PCs with the reflection peak at 520 nm and 590 nm were prepared; eventually, dropping the AIEgens-containing solution directly onto the PCs. AIEgens naturally deposits on PCs with the evaporation of the solvent ethanol. Since the periodic nanostructures of these PCs selectively reflect photons of a particular wavelength, they exhibit different colors. The structural color is closely related to the particle size of the microspheres. In a certain range, with the increase of particle size, the structural color gradually turns red. As shown in Figure 2a, PCs prepared using microspheres with different particle sizes have a smooth surface and uniform color distribution and PCs with a reflection peak of 520 nm shows a beautiful green color, and PCs with a reflection peak of 590 nm shows a bright red color.

### 2.2. Optical Characterization of PCs

PCs are attractive as a new type of spectral coding microcarriers. Their unique optical properties and unique nanostructures provide an effective means for biological and chemical analysis and have attracted extensive research interest in many fields. In particular, PCs with a fluorescence enhancement has great benefits, and controllable nanostructures have become promising materials for a range of bioanalytical applications. According to the size and dielectric properties of SiO_2_ NPs, different silica colloidal crystal beads each have a unique photonic band gap, which can inhibit the transmission of photons in a specific wavelength range, and then be reflected to produce a unique reflection spectrum [23]. As shown in Figure 2b, PCs exhibit a photonic band gap and a characteristic reflection peak. The peak position is based on its periodic structure, so the PCs are very stable and do not produce a controversial fluorescence signal. These characteristics make them suitable for multiple assays in different samples. We have calculated the reflection peak λ, λ = 1.633dn_average_ of the PCs according to Bragg’s law, where d is the center distance between two adjacent closed holes, and naverage is the average refractive index of the PCs structure. By calculation, the PCs reflection peaks prepared from the microspheres having particle diameters of 240 nm and 274 nm were 516 nm and 589 nm, which were highly consistent with the results in Figure 2b. PCs were characterized by scanning electron microscopy (SEM) images; the particle sizes were 240 nm and 274 nm, as shown in Figure 2c. During the solvent evaporation on the glass sheet, the SiO_2_ NPs self-assembled into a closely packed colloidal crystal array structure. The carbon scaffold also showed a fine ordered structure, which can be seen in the SEM image [24]. 

### 2.3. Fluorescence Enhancement Benefit of AIEgens

Immunoassay has become one of the most widely used tools because it is cost-efficient, time-saving, and user-friendly. It can detect certain biochemical substances, including small molecules, large molecules and pathogens. For example, Liu et al. [25] developed a molecularly imprinted polymers (MIPs)-based dual-modal ratiometric immunoassa to accurately diagnose hepatocellular carcinoma (HCC). Li’s team [26] developed an Aptamer-Linked CRISPR/Cas12a-Based Immunoassay, which realized the ultra-sensitive detection of biomarkers of different species. Therefore, the superior accuracy and sensitivity of immunoassays have prompted in-depth studies on many important issues. To improve the detection performance of immunoassay, a variety of optical sensing strategies have been proposed, including fluorometry, colorimetric method, and Raman spectroscopy. Among them, the fluoroimmunoassay method has attracted wide attention due to its easy operation and excellent stability. However, when the fluorescent substance concentration is too low, the LOD is not very low, making it difficult to complete the detection task [27]. However, when the fluorescent substance concentration is too high, fluorescence quenching occurs, which also reduces the detection sensitivity. Therefore, in order to solve this problem, we urgently need an excellent fluorescent substance. Since the first paper was published in 2001, AIEgens-based immunoassay has attracted much attention. Because the luminophor with AIE characteristics has excellent photostability, high quantum yield, excellent signal reliability and large stokes shift, it is more important that a high concentration of AIEgens does not undergo the fluorescence quenching phenomenon; therefore, this provides an optimal solution for the selection of the fluorescent substance [10]. 

**Figure 3:**
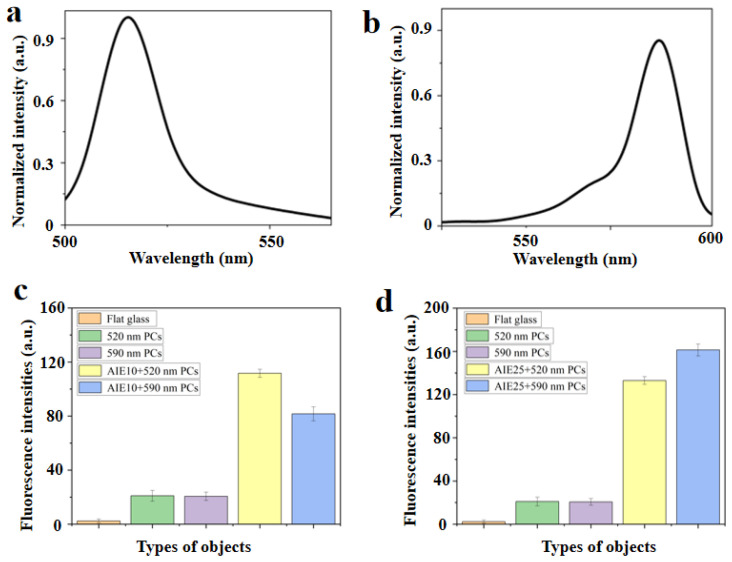
Emission spectra of (**a**) AIE10 and (**b**) AIE25 in ethanol. Fluorescence intensities of flat glass, PCs with different reflection peaks, and PCs incorporating (**c**) AIE10 and (**d**) AIE25.

Both AIE10 (Tetraphenyl ethylene-Br) and AIE25 (Tetraphenyl ethylene-NH_2_) are substances with aggregation-induced emissions. The chemical structures of AIE10 and AIE25 are shown in Figure 2d. Different fluorescent substances have their own particular maximum fluorescence emission wavelength. Figure 3a shows that AIE10 is excited by 365 nm light, and emits at a wavelength of 515 nm. Figure 3b shows that AIE25 is excited by 470 nm light, and emits at a wavelength of 580 nm. The higher the overlap between the maximum reflection peak of the PCs and the maximum fluorescence emission wavelength of the luminous source, the stronger the resulting fluorescence enhancement effect [28]. To verify the fluorescence enhancement efficiency of AIEgens, we repeated the testing of the fluorescence intensities of 520 nm PCs and 590 nm PCs in the absence of AIEgens and 520 nm PCs and 590 nm PCs when AIE10 and AIE25 were used as fluorescent substrates (AIE10 was excited with 365 nm light and AIE25 was excited with 470 nm light), respectively. By comparing these data with the fluorescence intensity of glass sheets, we found that when AIE10 was combined with 520 nm PCs, the fluorescence enhancement effect was the largest, which was four times that at 520 nm PCs in the absence of AIEgens. The enhanced effect is shown in Figure 3c. When AIE25 was combined with 590 nm PCs, the fluorescence enhancement was the largest, which was seven times that of 590 nm PCs in the absence of AIEgens. The enhanced effect is shown in Figure 3d.

### 2.4. Tumor Marker Detection

In clinical trials, high concentrations of AFP and CEA were found in cancer patients, including primary liver cancer, gastric cancer, intestinal cancer, and lung cancer [29]. Therefore, they are not specific to a particular type of cancer and are abundant in many types of cancer patients. Therefore, multiplex detection of AFP and CEA plays an important role in diagnosis, metastasis and prognosis.

**Figure 4:**
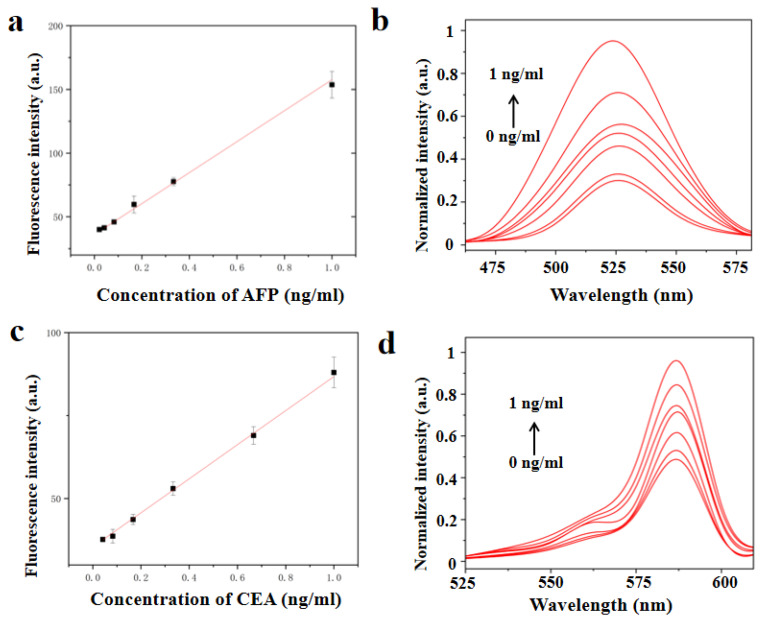
(**a**) calibration chart of AFP concentration and fluorescence intensity. (**b**) the variation of the Signal response with the concentration of AFP. (**c**) calibration chart of CEA concentration and fluorescence intensity. (**d**) the variation of the Signal response with the concentration of CEA. The error line represents the standard deviation of the three repeated measurements.

Herein, we chose AFP and CEA as typical multiple combinations of tumor markers for detection. Figure 4a shows the standard curve for an AFP bioassay based on AIE10-doped 520 nm PCs. The fluorescence intensity of AIEgens-doped PCs was determined by analyzing routine samples of AFP and CEA at concentrations ranging from 10 μg/mL to 1 ng/mL and plotted as a dose-response curves. Figure 4b shows the change of signal response with CEA concentration. The fluorescence intensity of the detection reservoir increased with the increase in AFP concentration. A good linear relationship between the fluorescence intensity and AFP concentration was observed within the detection range of 0.0416–1 ng/mL. The LOD, which was calculated as three times the standard deviation of the testing results of the blank divided by the slope of the calibration curve, was 0.0377 ng/mL. Figure 4c shows the standard curve of CEA bioassay based on AIE25-doped 590 nm PCs. The fluorescence intensity of the detection reservoir increased with the increase of CEA concentration [30]. In the detection range of 0.0416–1 ng/mL, a good linear relationship between fluorescence intensity and CEA concentration was observed, and the detection limit of CEA was 0.0337 ng/mL. Figure 4d shows the change of signal response with CEA concentration. The cut-off values of AFP and CEA in routine hospital clinical diagnosis range from 5 ng/mL to 8 ng/mL [22]. Therefore, our method is sufficient for practical application within the detection range.

### 2.5. Determination of Cross Reactivity

**Figure 5:**
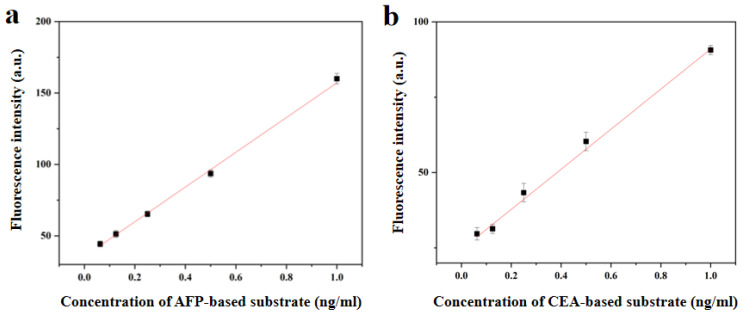
Fluorescence intensity function of (**a**) AFP mixed with a small amount of CEA and other interfering substances (**b**) CEA mixed with a small amount of AFP and other interfering substances drops with different concentrations on photonic crystals. The error line represents the standard deviation of the three repeated measurements.

Cross-reactivity will affect the reliability of multiplex immunoassay. In this study, we evaluated the cross-reactivity by detecting the fluorescence signals of substrates under a series of concentration gradients. It is the matrix AFP mixed with a small amount of CEA and other interfering substances (we call this mixture A+). Cross-reactivity plays an important role in the reliability of multiplex immunoassays. Figure 5a shows the standard curve of an A+ bioassay based on 520 nm PCs doped with AIE10. The fluorescence intensity of the detection cell increases with the increase of A+ concentration. The fluorescence intensity showed a good linear relationship with the concentration of A+ in the detection range of 0.0625–1 ng/mL, and the LOD A+ was 0.0514 ng/mL. Similarly, we also plotted the relationship between C+ and the fluorescence intensity of the concentration gradient. C+ consists of CEA mixed with a small amount of AFP and other impurities. As shown in Figure 5b, the fluorescence intensity of the detection cell increased with the increase in C+ concentration. The fluorescence intensity was linear over the range of 0.0625–1 ng/mL for C+ with the limit of detection 0.0452 ng/mL for C+. The results showed that the cross-reactivity between AFP and CEA was negligible and we were able to perform multiple immunoassays.

### 2.6. Practical Application of AIEgens-Doped PCs

**Figure 6:**
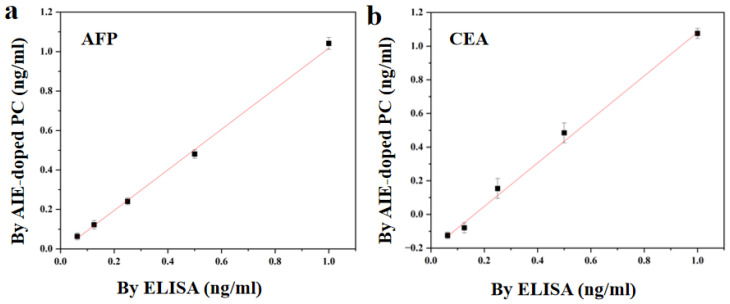
Correlation between multiplex bioassay based on AIEgens-doped PCs and ELISA for the determination of (**a**) AFP and (**b**) CEA in pbs samples. Any sample was repeated three times. The error line represents the standard deviation.

To evaluate the clinical potential and reliability of AIEgens-doped PCs, the method was compared to a method commonly used in clinical laboratories known as enzyme-linked immunosorbent assay (ELISA). We repeated the examination of five groups of pbs samples and the results are shown in Figure 6, with the regression equation (linear) as follows (x-axis, ECLIA; Y-axis, multiplex bioassay based on AIEgens-doped PCs):

Y = 1.0279x − 0.0107, R^2^ = 0.9968 (for AFP);

Y = 1.2929x − 0.2115, R^2^ = 0.9975 (for CEA).

These data show that our multiple bioassay based on AIEgens-doped PCs is very consistent with ELISA. This means that our method has good reliability and application potential. In addition, our multiple bioassay based on AIEgens-doped PCs can realize simultaneous detection on the same glass sheet, which is more flexible, time-saving and sample-saving than ELISA.

## 3. Conclusions

In summary, a fluorescent assay based on AIEgens-doped PCs has been developed for multiple detection of tumor markers. The PCs ensure that it has a certain fluorescence basis. The aggregation-induced luminescence benefit of AIEgens avoids the fluorescence quenching phenomenon caused by high fluorescence concentration, and greatly enhances the detection sensitivity. Therefore, in order to show better fluorescence enhancement benefit, we have carefully studied and optimized the reaction conditions and detection conditions by combining PCs with AIEgens, and found that multiple tumor markers (AFP and CEA) detection based on AIEgens-doped PCs showed high sensitivity and adequate detection range. In addition, in the multiple detection of actual pbs samples, compared with the ELISA method, this method has the same effect as the commonly used clinical detection methods. Conventional methods of detecting tumor markers using PCs typically have an LOD greater than 0.1 ng/mL [22], while our protocol had an LOD of 0.0377 ng/mL. By contrast, the FD method based on AIEgens-doped PCs was simple, rapid, stable and highly sensitive. These features make it an excellent way to detect malignant tumors. Therefore, our multiple biological assays based on AIEgens-doped PCs deserve further development and are expected to find applications in many fields, including fluorescence sensors, detection of skin diseases and fundus diseases.

## Data Availability

Since the data is also part of an ongoing study, it cannot be shared at this time.
